# Increased intestinal carbonate precipitate abundance in the sea bream (*Sparus aurata L*.) in response to ocean acidification

**DOI:** 10.1371/journal.pone.0218473

**Published:** 2019-06-21

**Authors:** Sílvia F. Gregório, Ignacio Ruiz-Jarabo, Edison M. Carvalho, Juan Fuentes

**Affiliations:** Centre of Marine Sciences (CCMar), Universidade do Algarve, Campus de Gambelas, Faro, Portugal; Universidade de Vigo, SPAIN

## Abstract

Marine fish contribute to the carbon cycle by producing mineralized intestinal precipitates generated as by-products of their osmoregulation. Here we aimed at characterizing the control of epithelial bicarbonate secretion and intestinal precipitate presence in the gilthead sea bream in response to predicted near future increases of environmental CO_2_. Our results demonstrate that hypercapnia (950 and 1800 μatm CO_2_) elicits higher intestine epithelial HCO_3_^-^ secretion *ex vivo* and a subsequent parallel increase of intestinal precipitate presence *in vivo* when compared to present values (440 μatm CO_2_). Intestinal gene expression analysis in response to environmental hypercapnia revealed the up-regulation of transporters involved in the intestinal bicarbonate secretion cascade such as the basolateral sodium bicarbonate co-transporter *slc4a4*, and the apical anion transporters *slc26a3 and slc26a6* of sea bream. In addition, other genes involved in intestinal ion uptake linked to water absorption such as the apical *nkcc2* and *aquaporin 1b* expression, indicating that hypercapnia influences different levels of intestinal physiology. Taken together the current results are consistent with an intestinal physiological response leading to higher bicarbonate secretion in the intestine of the sea bream paralleled by increased luminal carbonate precipitate abundance and the main related transporters in response to ocean acidification.

## Introduction

In seawater, fish live in a hyperosmotic environment with an osmolality ranging from 1000–1100 mOsm/kg which leads to a loss of body fluids. The water lost passively through epithelial surfaces by the gills and skin is compensated by increased drinking [1,2]. Due to the osmotic imbalance between the ingested seawater (SW) and the internal body fluids, an intense desalinization in the oesophagus is required [3, 4]. This ion absorption, mainly Na^+^ and Cl^-^, results in the decrease of intestinal fluid osmolality from circa 1050 to 400 mOsm kg^-1^ due to the action of active and passive transport processes [5–7]. The osmotic pressure of the intestinal fluid is then decreased to a level that matches plasma osmolality [8–10] allowing solute driven water absorption either paracellularly or through aquaporins [11,12].

Water absorption in the anterior part of the intestine of marine fish was long believed to be associated only with ion fluxes driven by Na^+^/K^+^/2Cl^-^ co-transporters [13]. However, it was recently established that part of the process is also linked to the action of chloride-dependent movements coupled to bicarbonate secretion, and mediated by apical anion transporters [14,15]. Members of the Slc26 family are responsible for this apical mechanism in fish enterocytes [10,16–19]. HCO_3_^-^ enters the cell through a basolateral Na^+^/HCO_3_^-^ co-transporter belonging to the Slc4 family [20,21] or is formed by the intracellular action of carbonic anhydrases that catalyze hydration of CO_2_ resulting in the formation of HCO_3_^-^ and H^+^ ions [22].

The secretion of HCO_3_^-^ into the intestinal luminal fluid promotes an increase of pH [23]. In parallel calcium and magnesium are present in high concentrations in SW and accumulate in the intestinal fluids as a consequence of water absorption. High divalent cations together with alkaline conditions promote precipitation of carbonate in the intestine of marine fish. As a result of this process, an osmolality reduction of the fluid occurs, thus facilitating water absorption [8,24]. Apical bicarbonate secretion in the enterocyte is regulated by biotic factors such as hormones, peptides, plasma HCO_3_^-^ concentration and abiotic factors *e*.*g*. salinity [10,25,26]. On the other hand, previous studies have shown that bicarbonate secretion in the intestine of marine fish is also regulated by the soluble adenylyl cyclase, an intracellular pH/CO_2_/HCO_3_^-^ sensor [27,28]. Moreover, a challenge with high environmental levels of CO_2_ (hypercapnia) elicited a response in the sea bream, decreasing its plasma pH during the initial 24 h, which is then buffered by increased levels of plasma bicarbonate [29]. Such an accumulation of bicarbonate in plasma, but also in other body fluids was also observed in several organisms, including marine fish [30,31]. Given the dependence of intestinal bicarbonate secretion on plasma/basolateral CO_2_/ HCO_3_^-^ levels [20,32], it should be of interest to establish if those physiological modifications alter intestinal carbonate precipitate formation in marine fish.

In a global scenario, as a result of anthropogenic emissions in the atmosphere CO_2_ increased from 280 up to 400 μatm in the 20^th^ century. The ocean absorbs part of this CO_2_ resulting in a decrease of pH [33,34]. The ocean surface CO_2_ levels are predicted to double by the year 2100 to achieve levels circa 1000 μatm CO_2_, thus decreasing 0.3–0.4 pH units [35–38]. Previous studies have reported that some marine calcifying biota are affected when transferred to elevated CO_2_ levels and the subsequent pH decrease close to values expected by the year 2100 (pH 7.8) and by the end of the next century (pH 7.6) [39–41]. Moreover, by the year 2300 the seawater CO_2_ concentration will be 1900 μatm that in turn will decrease the pH by up to 0.77 units [34,42–44]. Marine fish contribute to the marine inorganic carbon cycle as they produce up to 15% of new surface carbonate, as mineralized intestinal precipitates generated as by-products of their osmoregulation [24,45]. These carbonate precipitates are species-specific and vary not only on morphology but also have different carbonate phases that undergo different fates and roles in sediment generation and in the inorganic carbon cycle [46–48]. The nature of those precipitates makes them relevant as they provide neutralizing alkaline buffer in the form of soluble carbonates, in the most productive areas of the ocean [24,46]. Moreover, fish exposure to elevated CO_2_ scenarios [49,50] increase intestinal bicarbonate secretion as previously demonstrated in intestinal preparations and in intact animals [15,51,52].

Considering the importance of the process of intestinal bicarbonate secretion for both marine fish osmoregulation and the global ocean carbonate chemistry, the gilthead sea bream (*Sparus aurata*) has been the focus of our previous work on the regulation of intestinal bicarbonate secretion either in response to environmental challenges such as salinity [10] or the regulation by endocrine factors [18,27,32,53–56]. We hypothesized that prolonged periods of exposure to elevated CO_2_ would modify intestinal physiology and ion transporter function to protect plasma ionic homeostasis. Therefore, in the present study, we investigated intestinal bicarbonate secretion and carbonate abundance in response to long term acclimation to climate change relevant levels of CO_2_ combining whole animal measurements, with in vitro and molecular analyses.

## Methods

### Animals

Sea bream (*Sparus aurata*) juveniles were purchased from CUPIMAR SA (Cadiz, Spain) and transported to Ramalhete Marine Station (CCMAR, University of Algarve, Faro, Portugal). Fish were maintained for 60 days in 1000 L tanks with running seawater (36 ppt) at a density 9–10 kg m^-3^ and fed 2% ration (fish wet weight, Sorgal, S.A., Portugal; Balance 3) twice daily until the start of the experiment (all food was consumed by the fish). For experimental purposes, 5 fish (200 g body weight) were transferred to 100 L tanks without seawater re-circulating i.e., a continuous water flow-through. Temperature was maintained constant (25°C), photoperiod was natural (April-June, Algarve, Portugal) and the feeding regime was maintained as above. Food was withheld for 36 h before sacrifice and sampling to ensure complete absence of undigested food from the intestine. No mortality was observed during the experiments.

The experiments comply with the guidelines of the European Union Council (86/609/EU). All fish protocols were performed approved by the ethical committee ORBEA of CCMar-University of Algarve and performed under a ‘‘Group-C” license from the Direcção-Geral de Veterinária, Ministério da Agricultura, do Desenvolvimento Rural e das Pescas, Portugal.

### Experimental conditions and seawater chemistry

Each treatment (control, medium and high *p*CO_2_) had eight replicates (5 fish per tank).

The rate of CO_2_ injection into the systems was controlled by the pH level of seawater using pH probes connected to CO_2_ injection controllers (EXAxt PH450G; Yokogawa Iberia-Portugal) following guidelines provided by the manufacturer. Each independent header-tank was gassed with CO_2_, thus obtaining three groups constantly maintained at 440 (control), 950 (medium) and 1800 (high) μatm CO_2_. Seawater pH (NBS scale) was measured daily to calibrate the automated negative feedback system for CO_2_ injection. Based on the salinity, temperature, alkalinity and pH measurements we were able to calculate water *p*CO2 using CO2Calc.

Total alkalinity (TA) was measured using a combination DL15 titrator and a DG115-SC probe (Mettler-Toledo) using certified acid titrant (0.1 M HCl, Fluka Analytical, Sigma-Aldrich). Values of alkalinity, water pH (NBS scale), temperature (°C) and salinity were measured daily between 9 and 10.30 AM, and entered into CO2Calc Software (version 1.0.4) [57], using the constants K_1_ from [58] refit by Dickson and Miller [59], and [60] for KHSO_4_ to calculate pCO_2_.

### General sampling

After 3 months of acclimation to the altered water CO_2_ levels, fish were obtained in consecutive days and anesthetized in 2-phenoxyethanol (1: 10,000 v/v; Sigma- Aldrich, St Louis, MO, USA). Blood samples were collected by caudal puncture using heparinized syringes. Plasma was obtained by centrifugation (10,000 *rpm*, 5 min, 4°C) and stored at −20°C until analysis. Fish were sacrificed by decapitation and the whole intestine was isolated. The intestinal fluid of individual fish was collected from the excised intestinal tract clamped (from pyloric caeca to anal sphincter) with two haemostatic forceps, emptied into pre-weighed vials and centrifuged (12,000 *rpm*, 5 min, RT) to separate fluid from precipitates. The fluid was transferred to pre-weighed vials and the volume was measured to the nearest 0.1 μL (0.1 mg, assuming a density of 1).

### Plasma, fluid and precipitate analysis

Osmolality of plasma and intestinal luminal fluid was measured in 10 μL samples with a Vapro 5520 osmometer (Wescor, South Logan, UT, USA). Sodium concentrations were measured in duplicate in diluted samples (1:80 in milliq water) by flame photometry (BWB-XP Performance Plus, BWB Technologies, UK). The results are expressed as mmol L^-1^. Chloride was determined by coulometric titration (SAT-500, DKK-TOA, Japan). Calcium and magnesium were measured by colorimetric tests, using commercial kits (Spinreact, Reactivos Spinreact, SA, Girona, Spain), according to the manufacturer instruction in a microplate reader (Biorad Benchmark, Bio-Rad, USA). Intestinal fluid titratable alkalinity (HCO_3_^-^ + CO_3_^2-^) was manually measured with the double titration method with a pH electrode (HI1330B, Hanna Instruments, Smithfield, RI, USA) attached to a pH meter (PHM84, Radiometer, Copenhagen, Denmark): 50 μl of intestinal fluid samples was diluted in 10 mL NaCl (40 mmol L^-1^), gassed with CO_2_-free gas for 30 minutes to remove CO_2_ and titrated to pH 3.8 with 10 mmol L^-1^ HCl and additional gassing period of 20 minutes was applied to remove any remaining CO_2_. The sample was back titrated to its original pH with 10 mmol L^-1^ NaOH. The volume difference between added acid and base in both titrations and titrant molarities was used to calculate total HCO_3_^–^ equivalents (mEquiv.L^–1^) as described before [24,52,55]. Intestinal precipitates were re-suspended in 400 μl of triple-distilled water, homogenized in a glass homogenizer and a 100 μl aliquot was double titrated as described for the intestinal fluid, and normalized by fish mass and expressed as mEquiv/g body mass.

### Intestinal bicarbonate secretion *ex vivo*

A segment of fish anterior intestine was excised, mounted on tissue holders (P2413, 0.71 cm^2^, Physiologic Instruments, San Diego, CA, USA) and positioned between two half-chambers (P2400, Physiologic Instruments) containing 1.5 mL of serosal and mucosal saline. The composition of the serosal saline was: 160 mmol L^–1^ NaCl, 1 mmol L^–1^ MgSO_4_, 2 mmol L^–1^ NaH_2_PO_4_, 1.5 mmol L^–1^ CaCl_2_, 5 mmol L^–1^ NaHCO_3_, 3 mmol L^–1^ KCl, 5.5 mmol L^–1^ glucose and 5 mmol L^–1^ HEPES, pH 7.800, gassed with 0.3% CO_2_ + 99.7% O_2_. Mucosal saline: 88 mmol L^–1^ NaCl, 9.5 mmol L^–1^ MgCl_2_, 3 mmol L^–1^ KCl, 7.5 mmol L^–1^ CaCl_2_, 126.5 mmol L^–1^ MgSO_4_ and 1 mmol L^–1^ Na_2_HPO_4_, gassed with 100% O_2_ and pH maintained at 7.800 throughout the experiments by pH-Stat (see below). The temperature was maintained at 25°C throughout all experiments. All bioelectrical variables were monitored by means of Ag/AgCl electrodes (with tip asymmetry <1 mV) connected to either side of the Ussing chamber with 3 mm- bore agar bridges (1 mol L^-1^ KCl in 3% agar). Transepithelial electrical potential (TEP, mV) was monitored by clamping of epithelia to 0 μA cm^–2^. Epithelial resistance (Rt, Ω cm^2^) was manually calculated (Ohm’s law) using the voltage deflections induced by a 10 μA cm^–2^ bilateral pulse of 2 s every minute. Current injections were performed by means of a VCC 600 amplifiers (Physiologic Instruments). For pH-Stat control, a pH electrode (PHC 4000–8, Radiometer) and a micro-burette tip were immersed in the luminal saline and connected to a pH-Stat system (TIM 854, Radiometer). To allow pulsing (for Rt calculation) during pH measurements, the amplifier was grounded to the titration unit. The configuration of amplifier/pH-Stat system used in this study is similar to that first established for the characterization of HCO_3_^–^ secretion in the intestine of the Gulf toadfish and sea bream [10,20,61,62] and provides rates of intestinal secretion similar to those obtained by the double titration method. Measurement of HCO_3_^–^ secretion was performed on luminal saline at physiological pH 7.800 during all experiments. The volume of the acid titrant (2.5 mmol L^–1^ HCl) was recorded and the amount of HCO_3_^–^ secreted (nmol h^–1^ cm^–2^) was calculated from the volume of titrant added, the concentration of the titrant and the surface area (cm^2^). All experiments comprised 1 h of tissue stable voltage and HCO_3_^–^ secretion.

### qPCR

After anesthesia and decapitation, a portion of the anterior intestine was collected from individual fish, and stored in RNA Later at 4°C (Sigma- Aldrich) until utilized for RNA extraction within 2 weeks. Total RNA was extracted from samples of anterior intestine with the Total RNA Kit I (E.Z.N.A, Omega, US) following the manufacturer´s instructions and the quantity and quality of RNA assessed (Nanodrop 1000, Thermo Scientific, US). Prior to cDNA synthesis RNA was treated with DNase using the DNA-free Kit (Ambion, UK) following the supplier’s instructions. Reverse transcription of RNA into cDNA was carried out using the RevertAid First Strand cDNA Synthesis Kit (TermoFisher Scientific, UK) with 500 ng of total RNA in a reaction volume of 20 μL. Table 1 shows primer sequences and amplicon sizes.

**Table 1:** Details of primers used for qPCR.

Gene	Primer	Sequence (5´to 3´)	Tm (°C)	Product size(bp)	Efficiency (%)	NCBI accession no.
***slc26a3***	Forward	ATCTCGGCTCTGAAGGGACT	60	107	95	AM973894
	Reverse	AGCGAGCA TTTCTGTCCCTGCTC				
***slc26a6***	Forward	GCGGGACTGTTCAGCGGAGG	60	153	98	FM155691.1
	Reverse	TGCGAACACGCCTGAACGGCA				
***slc4a4***	Forward	ACCTTCA TGCCACCGCAGGG	60	101	97	FM157528.1
	Reverse	CGCCGCCGCCGATAACTCTT				
***nkcc2***	Forward	ACGGAGTCCAAGAAAACCACGGG	60	128	99	FP335045
	Reverse	CCAGCCAGGATTCCGGTCGC				
***aqp1a***	Forward	GGCTCTCACGTACGATTTCC	60	158	96	AY626939
	Reverse	TCTGTGTGGGACTATTTTGACG				
***18S***	Forward	AACCAGACAAATCGCTCCAC	60	139	97	AY993930
	Reverse	CCTGCGGCTTAATTTGACTC				

Real-time qPCR amplifications were performed in duplicate in a final volume of 10 μL with 5 μL SsoFast EvaGreen Supermix (Bio-Rad, UK) as the reporter dye, around 20 ng cDNA, and 0.3 μM of each forward and reverse primers. Amplifications were performed in 96-well plates using the *One-step Plus* sequence detection system (Applied Biosystems, California, USA) with the following protocol: denaturation and enzyme activation step at 95°C for 2 min, followed by 40 cycles. After the amplification phase, a temperature-determining dissociation step was carried out at 65°C for 15 s, and 95°C for 15 s. For normalization of cDNA loading, all samples were run in parallel using 18S ribosomal RNA [10,18,55,56,63]. To estimate efficiencies, a standard curve was generated for each primer pair from 10-fold serial dilutions (from 1 to 0.001 pg) of a pool of first-stranded cDNA template from all samples. Standard curves represented the cycle threshold value as a function of the logarithm of the number of copies generated, defined arbitrarily as one copy for the most diluted standard. All calibration curves exhibited correlation coefficients R^2^ > 0.98, and the corresponding real-time PCR efficiencies between 95 and 99%.

### Statistics

All results are shown as mean ± standard error of the mean (mean ± SEM). After assessing homogeneity of variance and normality, statistical analysis of the data was carried out by using one-way analysis of variance using CO_2_ concentration as a factor of variance, followed by the *post hoc* Bonferroni t-test (Prism 5.0 (version 5.0b), GraphPad Software for McIntosh). The level of significance was set at *p*< 0.05.

## Results

No mortality was observed in any of the experimental groups.

### Experimental conditions—seawater chemistry

The environmental condition values (temperature, salinity and pH) were measured twice a day in this study and the results are shown in Table 2. Temperature and salinity levels were maintained within 25.7–25.8°C and 36.0–36.2 ppt, respectively. No differences were described between CO_2_ treatments (Table 2). The partial pressure of CO_2_ and pH in the control treatment averaged 439 ± 29 μatm and pH 8.17 ± 0.03, in the medium water CO_2_ treatment group 986 ± 29 μatm and pH 7.87 ± 0.01, whereas in high water CO_2_ treatment it averaged 1856 ± 47 μatm and pH 7.62 ± 0.01. Total alkalinity was not significantly different between treatments ranging between 2344 and 2530 μmol kg^-1^ SW (Table 2).

**Table 2:** Chemical conditions of seawater where sea bream were kept under different CO_2_ concentrations over a three month period (daily measurements (90 days) of 8 tanks per treatment).

	440 μatm CO_2_	950 μatm CO_2_	1800 μatm CO_2_
**pH (NBS scale)**	8.17 ± 0.03	7.87 ± 0.01	7.62 ± 0.01
^*^pCO_**2**_ **(**μ**atm)**	439 ± 29	986 ± 29	1856 ± 47
**Salinity (ppt)**	36.2 ± 0.2	36.0 ± 0.1	36.2 ± 0.1
**Alkalinity (**μ**mol kg**^**-1**^ **SW)**	2455 ± 14	2450 ± 17	2447 ± 14
**T (°C)**	25.7 ± 0.3	25.8 ± 0.3	25.7 ± 0.2

* calculated using CO2cal (see methods).

### Plasma and intestinal fluid analysis

The osmolality and the ion composition of plasma and intestinal fluid of sea bream are shown in Tables 3 and 4, respectively. No differences were detected in plasma osmolality, which ranged between 343 and 346 mOsm kg^-1^ in controls and fish exposed to medium and high water CO_2_ concentrations (Table 3). Additionally, no changes in Cl^-^ (163.5 to 165.3 mmol L^-1^) and Na^+^ (178.7 to 186.2 mmol L^-1^) concentration (Table 3) were observed in the plasma between controls and fish exposed to medium and high water CO_2_ concentrations.

**Table 3:** Osmolality, Na^+^ and Cl^-^ levels in plasma of sea bream acclimated for 3 months to different CO_2_ concentration (440, 950 and 1800 μatm) at 36 ppt.

	440 μatm CO_2_	950 μatm CO_2_	1800 μatm CO_2_
**Osmolality (mOsm kg**^**-1**^**)**	342 ± 2	343 ± 2	346 ± 2
**Na**^**+**^ **(mmol L**^**-1**^**)**	178.7 ± 2.3	183.4 ± 2.6	186.2 ± 2.1
**Cl**^**-**^ **(mmol L**^**-1**^**)**	163.5 ± 1.3	165.2 ± 1.1	164.6 ± 1.3

Values are means ± SEM (N = 11–19). No differences were described between any of the experimental groups (p>0.05, one-way ANOVA).

**Table 4:** Osmolality, Na^+^, Ca^2+^ and Mg^2+^ levels in intestinal fluid of sea bream acclimated for 3 months to different CO_2_ concentration (440, 950 and 1800 μatm) at 36 ppt.

	440 μatm CO_2_	950 μatm CO_2_	1800 μatm CO_2_
**Osmolality (mOsm kg**^**-1**^**)**	353 ± 8	355 ± 7	359 ± 9
**Na**^**+**^**(mmol L**^**-1**^**)**	77.0 ± 2.4	72.1 ± 4.3	81.8 ± 5.0
**Ca**^**2+**^ **(mmol L**^**-1**^**)**	9.5 ± 0.6	10.3 ± 0.6	8.6 ± 0.5
**Mg**^**2+**^ **(mmol L**^**-1**^**)**	123.2 ± 4.3 ^a^	143.9 ± 7.8 ^b^	143.8 ± 6.5 ^b^

Values are means ± SEM (N = 8–18). Different letters indicate significant differences among groups (*p*<0.05, one-way ANOVA).

The content of Ca^2+^ (9.5 ± 0.6 mmol L^-1^) in intestinal fluid did not change with the different exposure to medium and high water CO_2_ concentrations (Table 4) and the concentration of Na^+^ (72.1 to 81.8 mmol L^-1^) in the intestinal fluid was not different between controls and fish exposed to medium and high water CO_2_ concentrations (Table 4).

**Fig 1:**
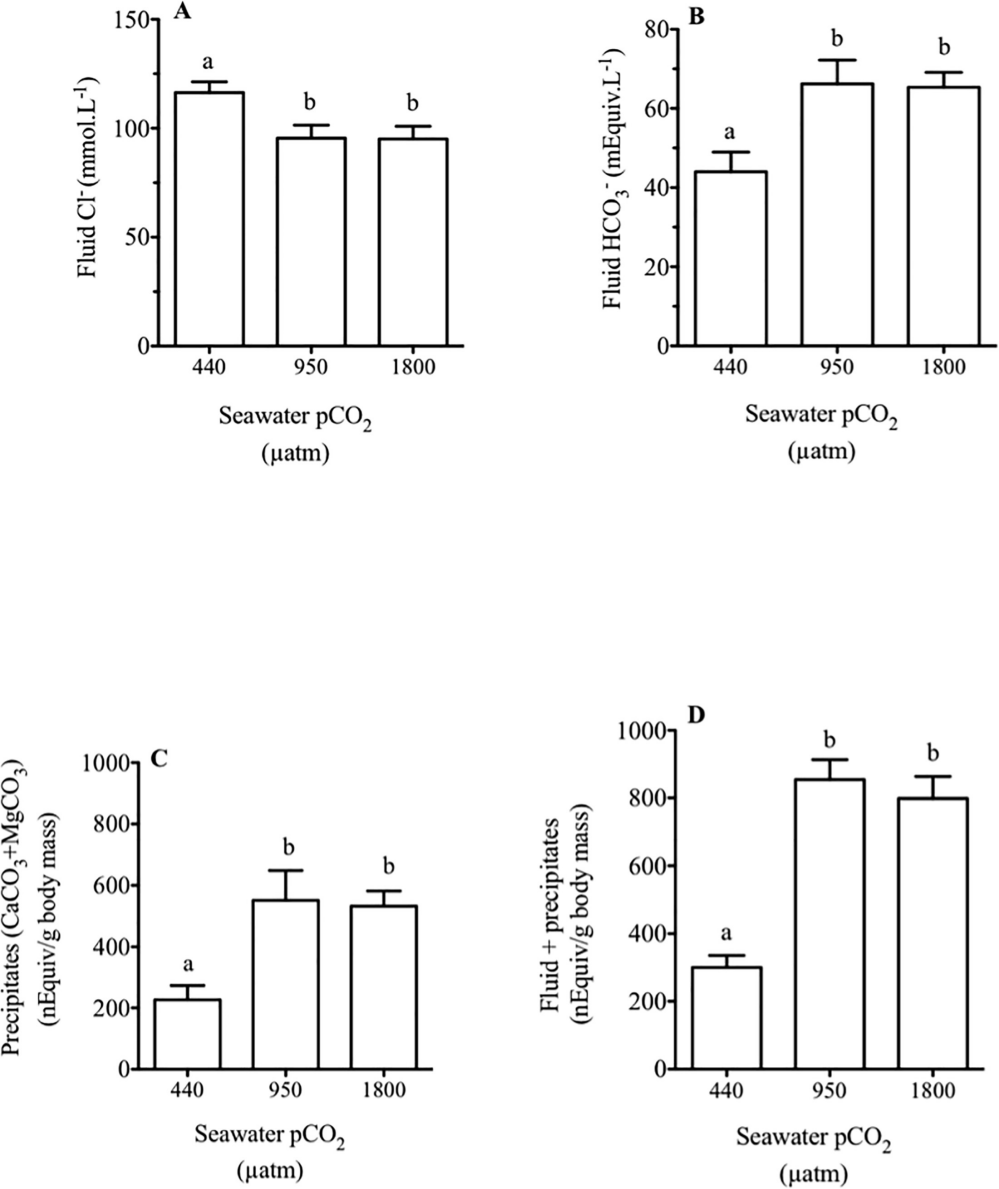
Characterization of intestinal fluid and precipitates of the sea bream. Characterization of Cl^-^ in intestinal fluid (A), HCO_3_^-^ content in intestinal fluid (B), Ca(Mg)CO_3_ precipitates (C) and total (fluid + precipitates) intestinal contents (D) of sea bream in response to different environmental CO_2_ levels (440, 950 and 1800 μatm CO_2_) for three months. Each bar represents the mean ± SEM (N = 7–9). Different superscript letters indicate significant differences (*p*<0.05, one-way ANOVA followed by Bonferroni *post hoc* test).

The content of Cl^-^ in the intestinal fluid of fish exposed to 950 and 1800 μatm CO_2_ was significantly lower 96.1 ± 4.7 mmol L^-1^ and 95.0 ± 5.3 mmol L^-1^ respectively (*p*<0.05, one-way ANOVA) when compared to controls 116 ± 5.5 mmol L^-1^ (Fig 1A). However, the content of Mg^2+^ in the intestinal fluid of fish exposed to 950 and 1800 μatm CO_2_ has increased significantly by 15% compared to the control fish (*p*<0.05, one-way ANOVA, Table 4).

### Bicarbonate and carbonate precipitates in the intestinal lumen

The normalized (μl/gram of fish mass) volume of fluid in the intestine of sea bream was in the range of 2.5 ± 0.4 in control fish and 2.4 ± 0.2 and 2.8 ± 0.2 (μl/gram) in medium and high water CO_2_ concentrations, respectively. HCO_3_^-^ concentration in the intestinal fluid of control fish was in the range of 44 mEq L^-1^ (Fig 1B) and the exposure to medium and high water CO_2_ concentrations resulted in a significant (*p*<0.05, one-way ANOVA) increase of HCO_3_^-^ concentration in the fish intestinal fluid to 66 and 65 mEq L^-1^ respectively (Fig 1B). When the total amount of bicarbonate equivalents was calculated for the whole contents of the intestine (i.e. fluid and precipitates) and fish body mass, we still observed that fish exposed to medium and high water CO_2_ concentrations responded with a 2.5-fold increase in carbonate precipitates in the intestine (*p*<0.05, one-way ANOVA, Fig 1C). A similar increase was observed in total of carbonates i.e. fluid and precipitates in intestines of fish exposed to medium and high water CO_2_ concentrations (*p*<0.05, one-way ANOVA, Fig 1D).

### Bicarbonate secretion *ex vivo*

**Fig 2:**
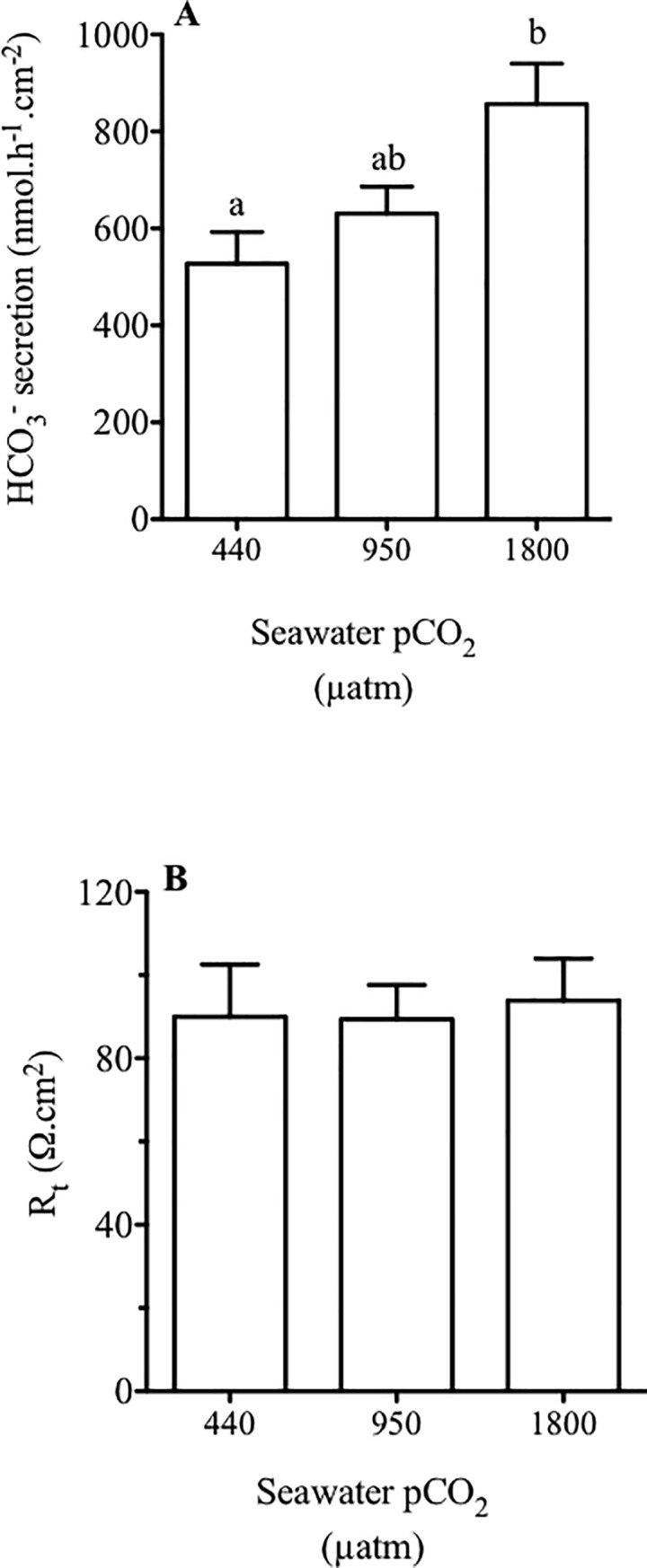
Bicarbonate secretion and tissue resistance measurements in the intestine of sea bream. Bicarbonate secretion (A) and tissue resistance (B) (Rt) measured *ex vivo* in Ussing chambers by pH-Stat from anterior intestinal regions of sea bream in response to different environmental CO_2_ concentrations (440, 950 and 1800 μatm CO_2_) for three months. Each bar represents the mean ± SEM (N = 5–7). Different superscript letters indicate significant differences (*p*<0.05, one-way ANOVA followed by Bonferroni *post hoc* test).

Bicarbonate secretion measured *ex vivo* in the anterior intestine is shown in Fig 2A. In control fish (440 μatm CO_2_) bicarbonate secretion was 511 ± 7.3 nmol.h^-1^.cm^-2^, while it averaged 631 ± 7.2 and 842 ± 6.9 nmol.h^-1^.cm^-2^ for fish acclimated to 950 (medium) and 1900 (high) μatm CO_2_, respectively. No significant effects were observed in bicarbonate secretion between the 440 and 950 μatm CO_2_ groups, while the 1800 μatm CO_2_-group increased from 38% in relation to the control fish. In terms of epithelial resistance, no changes were observed between groups (Fig 2B).

### qPCR

**Fig 3:**
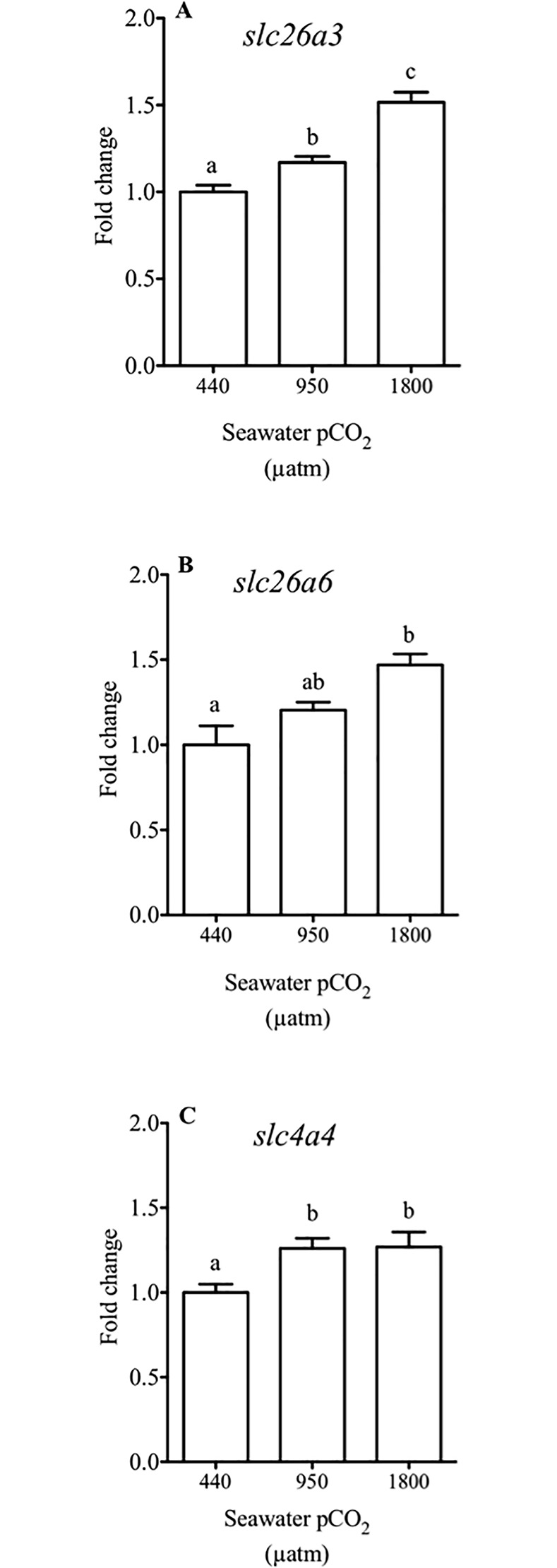
Relative expression of the genes in intestine from sea bream. Relative expression (fold change of gene expression using *18S* as the housekeeping gene) of *slc26a3* (**A**), *slc26a6* (**B**) and *slc4a4* (**C**) in the anterior intestine of sea bream in response to different environmental CO_2_ concentrations (440, 950 and 1800 μatm CO_2_) for three months. Each bar represents the mean ± SEM (N = 9–10). Different superscript letters indicate significant differences (*p*<0.05, one-way ANOVA followed by Bonferroni *post hoc* test).

The *slc26a3* anion exchanger had significant higher expression levels, around 20%, when fish were exposed to medium water CO_2_ concentrations and by about 50% to high water CO_2_ treatments (Fig 3A). The apical exchanger *slc26a6* presents a significantly higher expression, by about 45%, in fish exposed to high water CO_2_ levels compared to controls (*p*<0.05, one-way ANOVA, Fig 3B).

Expression of the basolateral co-transporter *slc4a4* was significantly (*p*<0.05, one-way ANOVA) higher in fish exposed to medium and high water CO2 concentrations, about 35% more than in control fish (Fig 3C).

**Fig 4:**
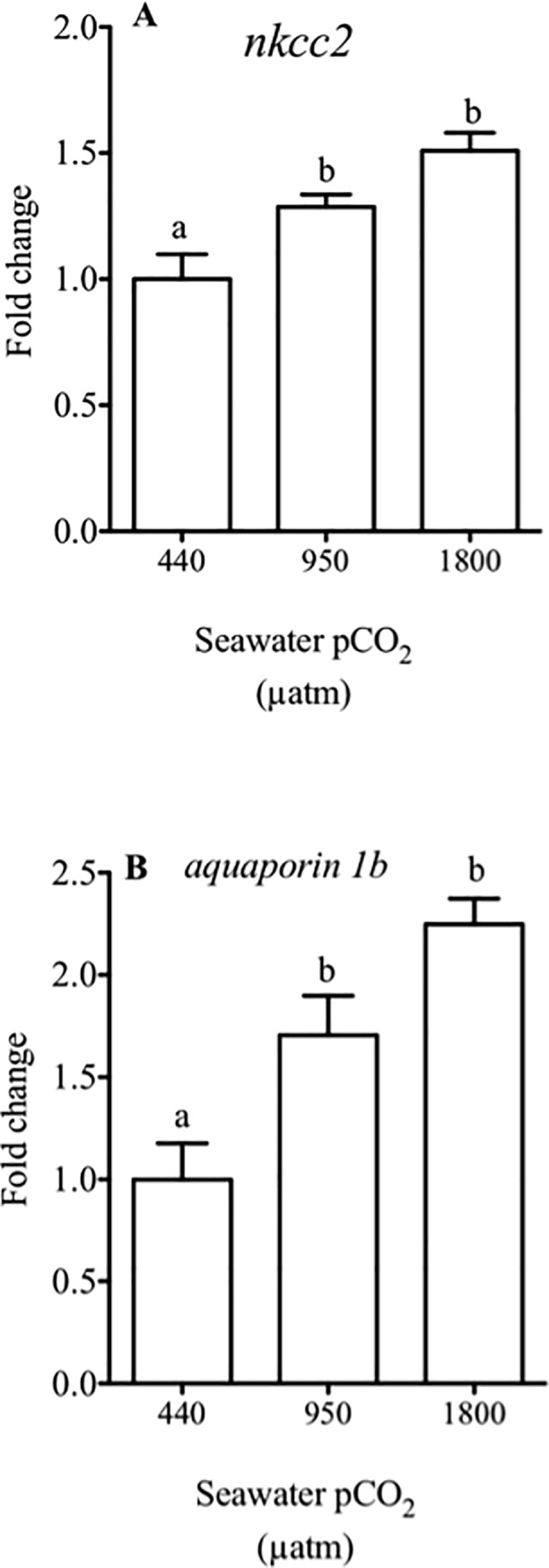
Relative expression of the genes in intestine from sea bream. Comparative analysis of fold change of gene expression in sea bream juveniles using qPCR of *nkcc2* and *aquaporin 1b* in the anterior intestine of sea bream in response to different CO_2_ concentrations (440, 950 and 1800 μatm CO_2_) for three months. Each bar represents the mean ± SEM (N = 8–11). Different superscript letters indicate significant differences (*p*<0.05, one-way ANOVA followed by Bonferroni *post hoc* test).

The expression of *nkcc2*, the absorptive form of the co-transporter in the anterior intestine of sea bream was significantly increased in response to hypercapnia, around 25%, when fish were exposed to medium and by about 50% to high water CO2 treatments (Fig 4A).

As expected, the water channel *aquaporin 1b* also responded positively to the CO_2_ challenge, with a 2 fold increase in fish exposed to high water CO_2_ concentrations than in controls (Fig 4B).

## Discussion

The sea bream, acclimated from 440 up to 1800 μatm CO_2_ for three months, is able to maintain within a narrow range its plasma levels of osmolality, Na^+^ and Cl^-^ without mortality. This homeostatic balance is partially achieved by changes in intestinal epithelia related to ion transport. In this regard, medium and high water CO_2_ concentration induces increased rates of epithelial HCO_3_^-^ secretion measured *ex vivo* and a molecular response that is in keeping with enhanced water absorption processes. Some results indicated that medium water CO_2_ (950 μatm) provide significant changes in bicarbonate transport pathways in the intestine.

Sea bream acclimated to elevated water CO_2_ for 3 months increased the amount of HCO_3_^-^ and carbonate precipitates in the intestinal fluid (Fig 1). This result is in good agreement with the data from the Gulf toadfish (*Opsanus beta*) exposed to 1900 μatm CO_2_ for 72 h that also shows an increase of 22.4% in intestinal bicarbonate secretion when compared with controls (380 μatm CO_2_) [15]. In our study, the groups of fish acclimated to hypercapnia (950 and 1800 μatm CO_2_), showed a 1.5-fold increase in the bicarbonate and 2.5-fold increase in the amount of carbonate precipitates present in the intestinal lumen when compared to the control group (440 μatm CO_2_). These results including the fold-change of variation, are in good agreement with those previously described by us in long term seawater-acclimated sea bream challenged with 55 ppt salinity [10]. These effects in relation to salinity challenge were related to an increase in water processing at the level of enterocytes, which also results in an enrichment of the intestinal fluid with Mg^2+^ and other divalent ions [11,16,25,26,64,65]. In the present study, an increase of Mg^2+^ in the order of 15% was shown in the intestinal fluid under high CO_2_ levels that was paralleled by the increase of carbonate precipitates, although it is unclear whether higher CO_2_ levels would alter the carbonate composition. The literature reports that Mg^2+^ content/proportion in the precipitated fraction is species dependent [48,66].We did not quantify the proportion of Mg^2+^ in the precipitates in response to high CO_2_ in the sea bream. However, considering the 2-fold increase of precipitates and the increase of Mg^2+^ in the fluid we could predict a proportional increase of Mg^2+^ in the precipitate of at least 15%.

In the intestinal fluid, both levels of hypercapnia elicited significant decreases in the intestinal fluid Cl^-^ content of about 21 mM. Interestingly, this decrease was paralleled by a ~22 mM increase in HCO_3_^-^ content of the same fluid, pointing to an activation of Cl^-^/ HCO_3_^-^ exchangers at the epithelial level [67]. To test this possibility, we measured bicarbonate secretion in the anterior intestine, since this portion of the intestine shows the highest secretion rates along the intestinal tract in the sea bream [10,27]. Accordingly, intestinal bicarbonate secretion increased in parallel with increased environmental CO_2_ levels (Fig 2) as was tested before in *Opsanus beta* [52]. The increases in measured bicarbonate secretion in the anterior intestine between the control group and fish challenged with 1800 μatm CO_2_ from 511 to 842 nmol HCO_3_^-^ cm^-2^ h^-1^, coincided well with the alterations reported in the intestine of the sea bream challenged long term with 55 ppt seawater, from 495 to 783 nmol HCO_3_^-^ cm^-2^ h^-1^ [10]. In this sense, the effects of hypercapnia could be carefully compared to a hyperosmotic challenge in this species, as they both enhanced bicarbonate secretion and abundance of carbonate precipitates in the intestine. A recent study in Gulf toadfish [51] exposed a 13% increase of intestinal HCO_3_^−^ secretion in fish challenged with high CO_2_ (1900 μatm) when analyzed with serosal saline mimicking changes in plasma chemistry. Here, the same serosal saline was used ex-vivo to measure bicarbonate secretion in Ussing chambers in all treatments. Therefore, while the increases in intestinal bicarbonate secretion in the present study are higher in the sea bream than in the toadfish, they represent an underestimation of actual secretion rates. This disparity in response amplitude between the toadfish [15,52] and the seabream (this study) could be related to calcium concentrations in intestinal fluids, which are higher in sea bream than in toadfish (> 2-fold). It is likely that luminal calcium functions as a limiting factor for precipitation in the intestine of the toadfish [17] and we have shown the calcium dependency of intestinal bicarbonate secretion in the sea bream [68]. In addition, it was previously reported that the sea bream responds to hypercapnia with a plasma pH drop, which is buffered within the first 5 days of exposure by increasing plasma bicarbonate levels [29]. We hereby suggest that the accumulation of plasma bicarbonate generated in response to elevated CO_2_, would be a causal factor for increased intestinal bicarbonate secretion at the level of the enterocytes [15,52,69]. Plasma bicarbonate enters the enterocyte through an electrogenic Na^+^-coupled HCO_3_^–^ transporter located at the basolateral membrane, *slc4a4*, which is important for a transepithelial HCO_3_^–^ transport [16,20]. Our results show that the expression of *slc4a4* increased in the groups challenged with high CO_2_ levels, supporting a hypothesis for enhanced bicarbonate secretion capacity in the intestine, in response to water hypercapnia and the consequent plasma HCO_3_^–^ accumulation.

In the apical membrane of the fish enterocytes, both bicarbonate secretion and chloride uptake are mediated through Slc26 family transporters, such as the *slc26a3 and slc26a6* [14,16,17,70]. Likewise those exchangers are expressed in the intestine of sea bream and are modulated by salinity and endocrine regulation [10,55]. In addition, the expression of these transporters is modulated in response to a CO_2_ challenge in the gill of toadfish [31]. Here, we confirmed that the expression of *slc26a6* and *slc26a3* are also modulated positively in response to high water CO_2_ levels in the intestine of the sea bream (Fig 3). This increase is in good agreement with the parallel expression increase of the *slc4a4* co-transporter, which would substantiate a boosted secretory flow of bicarbonate from the plasma to the intestinal lumen, subsequently enhancing chloride absorption. It is challenging to establish transporters stoichiometry from ion concentration alone. However, here we have established a 1:1 relationship between the variation of Cl^-^ and HCO_3_^-^ in the intestinal fluid after 3 months in hypercapnia (Fig 1A and 1B). It appears, that as a consequence of the increased bicarbonate levels on the intestinal fluid, carbonate presence is increased, sustaining the decrease of Cl^-^ in intestinal fluid required to enable water absorption [8].

In marine fish, water absorption in the intestinal tract is mediated through the activity of ion absorption, mainly Na^+^ and Cl^−^. One of the most important ion transporters in these species is the *Na*^*+*^*-K*^*+*^*-2Cl*^*-*^ *co-transporter* (*nkcc*) [13], which mediates the electroneutral movement of 1 Na^+^, 1 K^+^ and 2 Cl^-^ across cell membranes. Two isoforms, *nkcc1* and *nkcc2*, are currently known and are encoded by different genes [71]. In the intestine of marine fish the apical co-transporter *nkcc2* activity seems essential for ion regulation and, in parallel with the *slc26a6*, it is believed to regulate water homeostasis in salinity-challenged sea bream [10,17,61]. Here, we show that elevated water CO_2_ causes an up-regulation of *nkcc2* expression in the anterior intestine (Fig 4A), which adds up to increased expression of the apical anion transporters, *slc26a6* and *slc26a3*. This combination has the potential to enhance intestinal water absorption linked to chloride movements. Sea bream exposed to elevated water CO_2_ showed no significant changes in plasma ions concentration, indicating that the intestinal response to elevated water CO_2,_ is part of the allostatic control of plasma homeostasis. In order to understand these changes further, we analysed the expression of *aquaporin 1b*, a functional water channel highly expressed in intestinal epithelial cells of the sea bream anterior intestine [72,73]. We observed an increase of *aquaporin 1b* expression in the anterior intestine of sea bream exposed to elevated water CO_2_. Providing further evidence of the functional and molecular changes of the intestine in response to elevated water CO_2,_ that could be the foundation of increased energetic expenditure recently reported in the toadfish intestine in response to elevated water CO_2_ [51].

Fish aggregates are relevant to the ocean carbon cycle [50]. Here we show that fish exposed to future relevant levels of water CO2 predicted for ocean acidification have an intestinal molecular and functional response in acid-base regulation and osmoregulation consistent with increased in carbonate aggregates abundance. It will be essential to confirm if this increase in intestinal aggregates in response to ocean acidification will have a global impact on water chemistry.
